# Time-Course Transcriptomics Analysis Reveals Molecular Mechanisms of Salt-Tolerant and Salt-Sensitive Cotton Cultivars in Response to Salt Stress

**DOI:** 10.3390/ijms26010329

**Published:** 2025-01-02

**Authors:** Hang Li, Li Liu, Xianhui Kong, Xuwen Wang, Aijun Si, Fuxiang Zhao, Qian Huang, Yu Yu, Zhiwen Chen

**Affiliations:** 1 Cotton Institute, Xinjiang Academy of Agricultural and Reclamation Science/Northwest Inland Region Key Laboratory of Cotton Biology and Genetic Breeding, Shihezi 832000, China; lhup1997@163.com (H.L.); cottonliuli@sina.com (L.L.); kxh920@sohu.com (X.K.); wxw629@163.com (X.W.); siaijun1002@163.com (A.S.); 1365100845@163.com (F.Z.); hq10200102@163.com (Q.H.); 2 Engineering Research Center of Coal-Based Ecological Carbon Sequestration Technology of the Ministry of Education, Key Laboratory of Graphene Forestry Application of National Forest and Grass Administration, Shanxi Datong University, Datong 037009, China

**Keywords:** cotton, salt stress, comparative transcriptomics, phytohormone, salt-tolerant, salt-sensitive

## Abstract

Salt stress is an environmental factor that limits plant seed germination, growth, and survival. We performed a comparative RNA sequencing transcriptome analysis during germination of the seeds from two cultivars with contrasting salt tolerance responses. A transcriptomic comparison between salt-tolerant cotton cv Jin-mian 25 and salt-sensitive cotton cv Su-mian 3 revealed both similar and differential expression patterns between the two genotypes during salt stress. The expression of genes related to aquaporins, kinases, reactive oxygen species (ROS) scavenging, trehalose biosynthesis, and phytohormone biosynthesis and signaling that include ethylene (ET), gibberellin (GA), abscisic acid (ABA), jasmonic acid (JA), and brassinosteroid (BR) were systematically investigated between the cultivars. Despite the involvement of these genes in cotton’s response to salt stress in positive or negative ways, their expression levels were mostly similar in both genotypes. Interestingly, a *PXC2* gene (*Ghir_D08G025150*) was identified, which encodes a leucine-rich repeat receptor-like protein kinase (LRR-RLK). This gene showed an induced expression pattern after salt stress treatment in salt-tolerant cv Jin-mian 25 but not salt-sensitive cv Su-mian 3. Our multifaceted transcriptome approach illustrated a differential response to salt stress between salt-tolerant and salt-sensitive cotton.

## 1. Introduction

Cotton is a natural, high-quality fiber source and an important oil crop, which has a very important role in China’s economic development [1]. However, in recent years, there has been a rapid increase in the population, leading to an increasing demand for cotton, which has resulted in the expansion of the cotton cultivation scale [2]. The contradiction between “competing for land between cotton and grain” has become increasingly severe [3]. Human activities and global climate change have made soil salinization a more serious issue, with the global area of saline land reaching 9.5 × 10^8^ hm^2^ and growing by more than 1 million hectares per year [4]. Currently, China is severely affected by soil salinization, with the total area of saline soil estimated at around 99 million hectares, and soil salinization is one of the important factors affecting the cotton yield and quality [5], seriously affecting agricultural production and social and economic development. Screening and cultivating salt-tolerant cotton varieties is one of the most economical and effective ways to utilize salinized soil. By transferring cotton cultivation to saline and barren land, further expanding the cotton planting area, it has great significance for the cotton industry and economic development.

Cotton is a relatively salt-tolerant crop and a pioneering crop for saline land improvement, but its salt tolerance varies among the developmental stages and tissues of different genotypes [6]. High salt stress inhibits cotton growth and reduces the cotton yield and fiber quality [7]. Soil salt stress reduces the vitality of cotton root systems and the root water absorption ability, inhibiting water transport from roots to leaves. In the NaCl-simulated saline stress treatment, the increase in the Cl^−^, K^+^, Ca^2+^, and N content in leaves is positively correlated with cotton tolerance [7]. In addition, the activities of antioxidant enzymes, enzymes involved in secondary metabolism, active oxygen content, osmotic adjustment substance content, and plant hormones play important roles in regulating cotton’s response to salt stress [8]. Researchers have found that in saline stress treatment, salt-tolerant cotton cultivars are compared with salt-sensitive cultivars, with higher activities of antioxidant enzymes and enzymes involved in secondary metabolism, lower active oxygen content, and higher osmotic adjustment substances (such as proline) and secondary metabolite content [9]. Salt stress causes an ion imbalance in plant tissues, with salt-sensitive cultivars accumulating more Na^+^ but less K^+^ in their leaves compared to salt-tolerant cultivars. In contrast, the changes in the ion homeostasis of salt-tolerant cultivars are not obvious, and the K^+^/Na^+^ ratio increases [10]. Salt stress leads to changes in the content of fatty acids and hormones in cotton leaves, such as an increase in palmitic acid, stearic acid, and oleic acid, and a decrease in linoleic acid and linolenic acid [11]. Therefore, comprehending the molecular mechanism of salt tolerance in cotton and exploring key salt-tolerance genes is crucial for breeding resilient, salt-tolerant cotton cultivars.

Currently, with the completion of cotton genomic sequencing and assembly, the advancement of cotton functional genomics has been accelerated [12,13,14,15,16]. Based on the genomic data, transcriptomics has also made some progress in revealing the mechanisms of cotton salt tolerance and candidate gene identification. Transcriptomics has revealed some important genes that respond to salt stress in cotton, including membrane receptor proteins, transporters, transcription factors, CDPK, MAPK signal cascades, and hormone biosynthesis and signal transduction [17,18]. In the diploid cotton species *Gossypium davidsonii* with superior stress tolerance, transcriptome data elucidated that the salt overly sensitive (SOS) and ROS signaling pathways were closely related. Furthermore, photosynthesis pathways and metabolism play important roles in ion homeostasis and the oxidation balance in salt stress tolerance. Comparative transcriptome sequencing analysis was conducted on two landrace cotton cultivars with different salt tolerance, and it was found that some biological processes, such as transcriptional regulation, signal transduction, and secondary metabolism, exist in a markedly different manner between the two cultivars, providing scientific evidence explaining the differences in salt tolerance between the two cultivars [19]. Integrative transcriptomic analyses revealed that the ion transport, hormone metabolism, and ROS-scavenging pathways played important roles in cotton root adaptation to salt stress [20]. Using single-cell transcriptomics technology to systematically analyze the response of cotton roots to salt stress revealed that the differentially expressed genes (DEGs) identified were concentrated on the plant-type primary cell wall biogenesis, defense response, phenylpropanoid biosynthesis, transcription factors, and plant hormone metabolic pathways [21]. These results compared the transcriptomic expression profiles of cotton seedlings exposed to salt stress using RNA sequencing and revealed that cotton has different regulatory mechanisms in response to salt stress.

Genetic engineering and expressing of stress-resistant genes in cotton have also been proven to be an effective method for improving cotton salt stress resistance [22]. For instance, overexpressing the rice stress-resistant gene *SNAC1* in cotton can significantly improved the transgenic cotton’s resistance to salt stress [23]. Overexpressing the *Artemisia vulgaris* stress response gene *AvDH1* in cotton can reduce the damage caused by salt stress and increase the productivity in saline fields [24]. The *GhDof1* DOF transcription factor gene of the cotton family was induced to express under salt stress, and overexpressing *GhDof1* enhanced cotton’s resistance to salt stress [25]. Transgenic *ApGSMT2g* and *ApDMT2g* cotton showed higher salt tolerance and more seed cotton yield in saline fields compared to the wild type [26]. The bZIP family transcription factor participated in the regulation of cotton’s resistance to salt stress, and overexpressing the *GhABF2* and *GhABF3* genes in cotton improved its tolerance to salt stress by regulating the expression of some stress-related genes and increasing the activity of antioxidant enzymes [27,28]. These results demonstrated that using biotechnology to manipulate key candidate genes may be an important strategy for enhancing the stress resistance of cotton.

Despite these efforts, the regulatory molecular network of salt tolerance genes in cotton remains unclear. Furthermore, to the best of our knowledge, there has been no time-course comparative transcriptome analysis of the effect of salt stress in cotton. In this study, we explored the transcriptional responses of salt-tolerant and salt-sensitive cotton cultivars to salt stress. We aimed to compare the transcriptional responses associated with phytohormone signaling, regulation of gene expression related to aquaporins, kinases, and ROS scavenging, and trehalose metabolism following salt stress in two contrasting cotton varieties at different seed germination stages to better understand the mechanism involved in the response to salt stress. Our findings revealed temporal and genotype-specific responses to salt stress and identified critical salt-tolerant metabolic pathways and promising candidate genes for the engineering of salt-tolerant cotton.

## 2. Results

### 2.1. Salt Sensitivity Assessment of Su-Mian 3 and Jin-Mian 25 Cotton Cultivars

**Figure 1:**
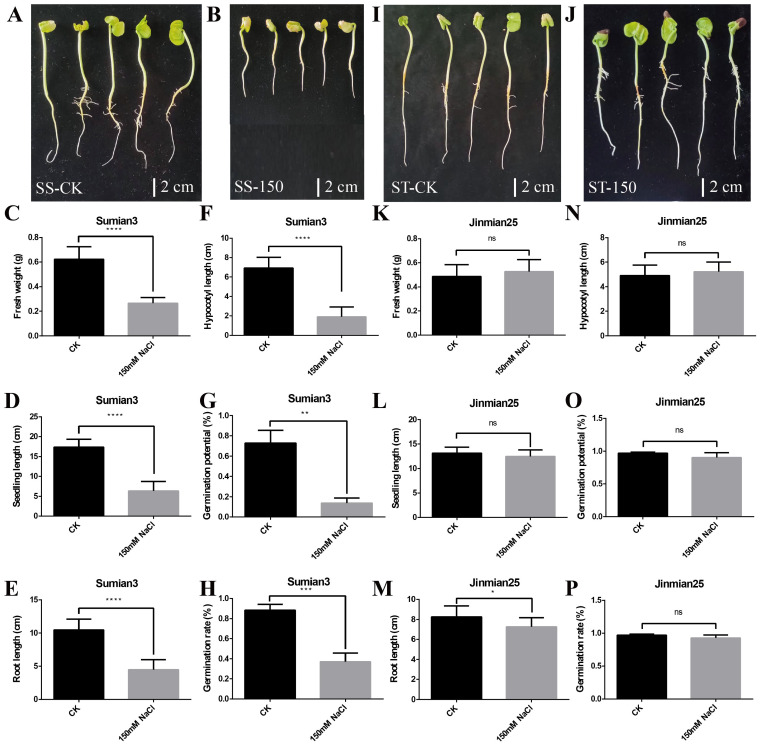
Salt sensitivity assessment of Su-mian 3 and Jin-mian 25 cotton plants. (**A**) The phenotype of Su-mian 3 (SS) seedlings grown in distilled water (CK) for seven days. (**B**) The phenotype of Su-mian 3 (SS) seedlings grown in 150 mM NaCl solution (150) for seven days. (**C**) Fresh weight of Su-mian 3 seedlings between CK and salt stress. (**D**) Length of Su-mian 3 seedlings between CK and salt stress. (**E**) Root length of Su-mian 3 seedlings between CK and salt stress. (**F**) Hypocotyl length of Su-mian 3 seedlings between CK and salt stress. (**G**) Germination potential of Su-mian 3 seeds. (**H**) Germination rate of Su-mian 3 seeds. (**I**) The phenotype of Jin-mian 25 (ST) seedlings grown in distilled water (CK) for seven days. (**J**) The phenotype of Jin-mian 25 (ST) seedlings grown in 150 mM NaCl solution (150) for seven days. (**K**) Fresh weight of Jin-mian 25 seedlings between CK and salt stress. (**L**) Length of Jin-mian 25 seedlings between CK and salt stress. (**M**) Root length of Jin-mian 25 seedlings between CK and salt stress. (**N**) Hypocotyl length of Jin-mian 25 seedlings between CK and salt stress. (**O**) Germination potential of Jin-mian 25 seeds. (**P**) Germination rate of Jin-mian 25 seeds. Note: Su-mian 3 is a salt-sensitive cultivar (SS-Salt Sensitive), and Jin-mian 25 is a salt-tolerant cultivar (ST-Salt Tolerant). Values are the mean ± SD, *n* = 5, * *p* < 0.05, ** *p* < 0.01, *** *p* < 0.001, **** *p* < 0.0001, ns: not significant. Student’s *t*-test.

The seeds of Su-mian 3 and Jin-mian 25 cotton were treated using distilled water and 150 mM NaCl solution. The germination potential and germination rate of the seeds, as well as the fresh weight, seedling length, root length, and hypocotyl length of the seedlings, were determined after 7 days of treatment (Figure 1). As shown in Figure 1, there was a significant difference in the salt tolerance testing between the two cotton cultivars. Compared to the control (Figure 1A), the growth of the Su-mian 3 seedlings was severely inhibited (Figure 1B), with the fresh weight (Figure 1C), seedling length (Figure 1D), root length (Figure 1E), and hypocotyl length (Figure 1F) significantly reduced by about 58%, 63%, 58%, and 73%, respectively. Meanwhile, the germination potential (Figure 1G) and germination rate (Figure 1H) of the Su-mian 3 seeds were significantly decreased by about 86% and 58%. However, Jin-mian 25 grew well under 150 mM salt stress treatment, and there was no obvious difference in the growth of the seedlings (Figure 1I,J). Overall, only the root length was significantly reduced by about 12% (Figure 1M), and the other indicators, including the fresh weight (Figure 1K), seedling length (Figure 1L), hypocotyl length (Figure 1N), germination potential (Figure 1O), and germination rate (Figure 1P), were hardly affected by salt stress and showed no significant differences between the control and salt treatment. The above results indicated that Su-mian 3 was a salt-sensitive (SS) cultivar, while Jin-mian 25 was a salt-tolerant (ST) cultivar.

### 2.2. Transcriptome Profiles of Su-Mian 3 and Jin-Mian 25 Cotton Plants Exposed to Salt Stress

**Figure 2:**
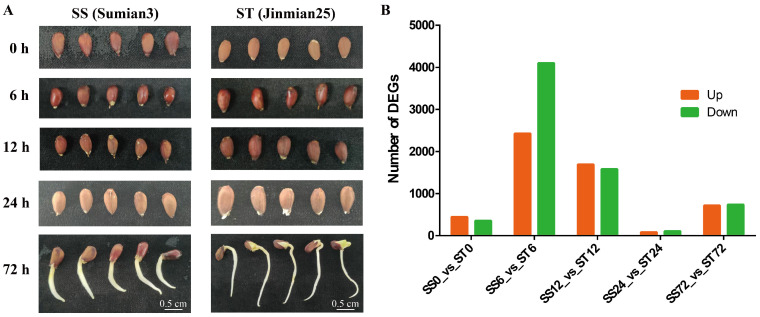
Seeds of cv Su-mian 3 and Jin-mian 25 treated with 150 mM NaCl solution for 0, 6, 12, 24, and 72 h for expression profile analyses and identification of DEGs. (**A**) Seed germination and seedling growth of cv Su-mian 3 and Jin-mian 25 under salt stress. (**B**) Number of DEGs between the comparisons of the SS0 vs. ST0, SS6 vs. ST6, SS12 vs. ST12, SS24 vs. ST24, and SS72 vs. ST72 groups.

To study how cv Su-mian 3 and Jin-mian 25 cotton plants respond to salt stress at the gene expression level, we analyzed the transcriptome profiles of the salt-sensitive cotton cv Su-mian 3 and the salt-tolerant cotton cv Jin-mian 25 (Figure S1). It is known that seed germination and seedling growth are the stages most affected by salt stress [29]. Hence, cv Su-mian 3 and Jin-mian 25 plants at the seed germination and seedling growth stages were used for the transcriptome profiling analyses. The seeds were completely submerged in 150 mM NaCl solution for 0, 6, 12, 24, and 72 h, and then sampled for expression profile analyses in response to salt stress (Figure 2A). On average, 27,002,000 paired-end reads were generated per sample, resulting in a total of 243 Gb of high-quality clean bases obtained from the 30 samples (Table S1). Using the *G. hirsutum* cv TM-1 reference genome [12], the average rates of mapped reads for the cv Su-mian 3 and Jin-mian 25 samples were 92.70% and 92.08%, respectively (Table S2).

As these two cultivars showed great variance in the response to salt stress, we evaluated the gene expression at five time points (0, 6, 12, 24, and 72 h) during salt treatment from cv Su-mian 3 and Jin-mian 25, hereafter referred to as SS0, SS6, SS12, SS24, SS72, ST0, ST6, ST12, ST24, and ST72, respectively. A total of 12,237 DEGs were identified across five comparisons: SS0 vs. ST0, SS6 vs. ST6, SS12 vs. ST12, SS24 vs. ST24, and SS72 vs. ST72 (Figure 2B). Specifically, 444 genes were significantly upregulated in ST0 compared to SS0, while 354 genes were significantly downregulated. In comparing ST6 with SS6, 2428 genes were significantly upregulated and 4100 genes were significantly downregulated. Furthermore, in the comparison between ST12 and SS12, 1691 genes were significantly upregulated and 1580 genes were significantly downregulated. On the other hand, when compared to the SS24 group, the ST24 group exhibited 83 upregulated genes and 105 downregulated genes. Similarly, when compared to the SS72 group, the ST72 group had 715 upregulated genes and 737 downregulated genes (Figure 2B).

### 2.3. Pathway Enrichment of DEGs

**Figure 3:**
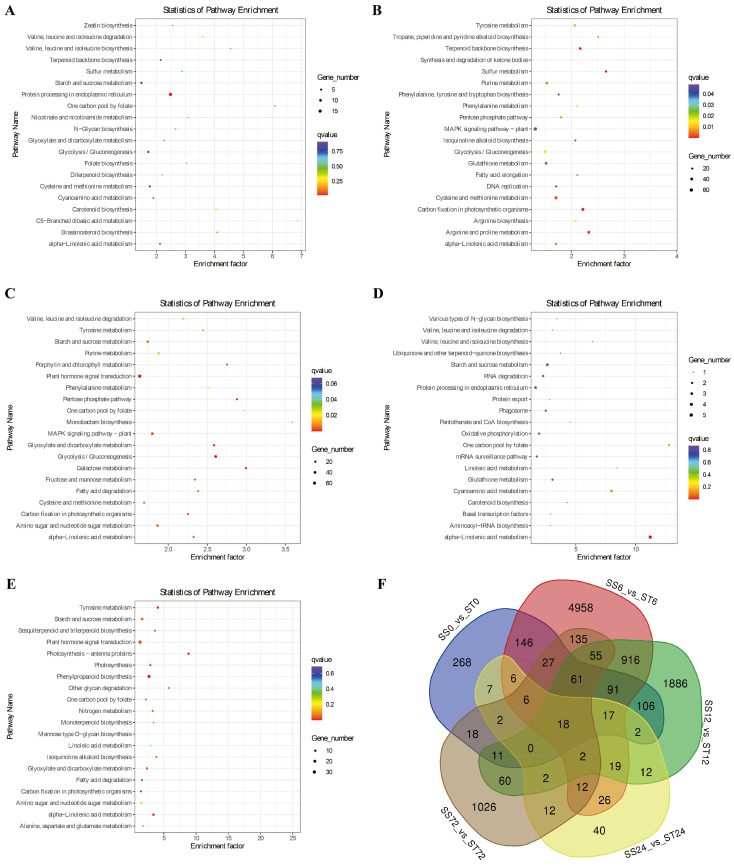
KEGG pathway analysis of enriched DEGs. (**A**) Top 20 pathways of significantly enriched DEGs from SS0 vs. ST0. (**B**) Top 20 pathways of significantly enriched DEGs from SS6 vs. ST6. (**C**) Top 20 pathways of significantly enriched DEGs from SS12 vs. ST12. (**D**) Top 20 pathways of significantly enriched DEGs from SS24 vs. ST24. (**E**) Top 20 pathways of significantly enriched DEGs from SS72 vs. ST72. (**F**) Venn diagram comparison of the DEGs from five time points after 150 mM NaCl stress between the two cotton cultivars.

Next, we conducted a KEGG pathway enrichment analysis to investigate the biological functions of these DEGs during salt stress in both genotypes. The results showed that these DEGs were mainly involved in protein processing in the endoplasmic reticulum, starch and sucrose metabolism, plant–pathogen interaction, plant hormone signal transduction, glyoxylate and dicarboxylate metabolism, glycolysis/gluconeogenesis, MAPK signaling pathway-plant, cysteine and methionine metabolism, and alpha-linolenic acid metabolism (Figure 3A–E), suggesting that these metabolic pathway genes might confer the resistance to salt stress in cv Jin-mian 25 cotton.

**Figure 4:**
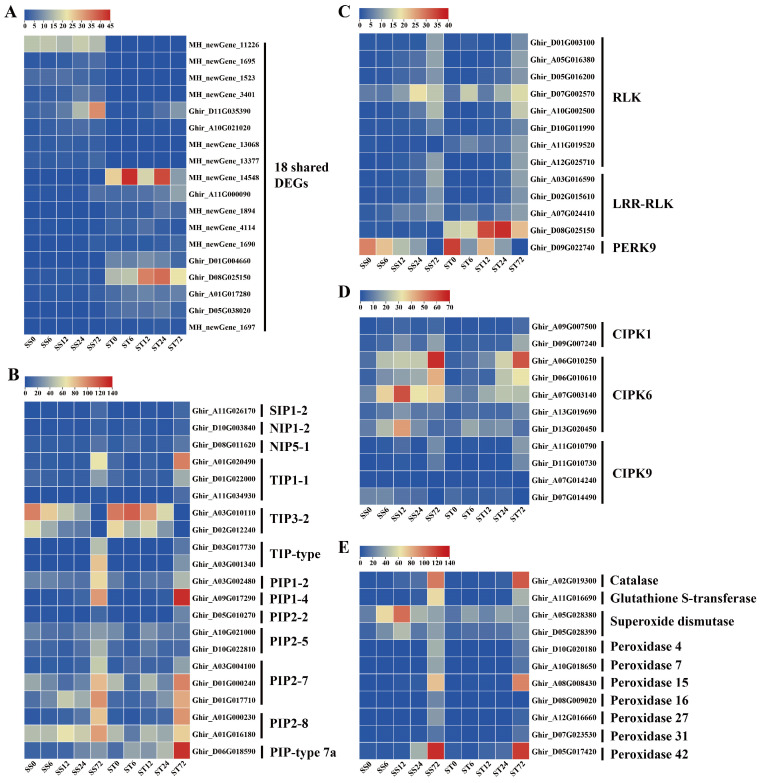
Expression patterns of different pathway genes during salt stress in both genotypes of cotton. (**A**) Shared DEGs of the two cotton cultivars from five time points after salt stress. (**B**) Aquaporin genes. (**C**) RLK genes. (**D**) CIPK genes. (**E**) ROS-scavenging enzyme genes.

Additionally, the Venn diagram showed that there were 18 shared DEGs between the two genotypes across the five time points (Figure 3F). Out of these 18 shared DEGs, 8 genes had their expression levels suppressed by salt stress, and 10 genes had their expression induced (Figure 4A). This includes the PXC2 gene (Ghir_D08G025150), which encodes the leucine-rich repeat receptor-like protein kinase (LRR-RLK). This gene showed an induced expression pattern at the 12, 24, and 72 h time points after salt stress treatment in only cv Jin-mian 25, with higher transcripts compared to cv Su-mian 3 (Figure 4A). This finding suggests that further functional verification of this gene is needed.

### 2.4. Expression Patterns of Genes Related to Aquaporins, Kinases, and ROS Scavenging During Salt Stress in Both Genotypes

To further mine the DEGs related to salt stress, we conducted a search for relevant pathways in databases using the keywords “salt” and “salt stress”, which helped us narrow down the list of candidate genes. The pathways we looked at included the following: response to salt stress (GO:0009651), negative regulation of response to salt stress (GO:1901001), regulation of response to salt stress (GO:1901000), cellular response to salt stress (GO:0071472), positive regulation of response to salt stress (GO:1901002), and response to salt (GO:1902074) from the GO database, as well as the MAPK signaling pathway-plant from the KEGG database. By using this approach, we were able to reduce the number of identified DEGs related to salt stress to 347.

In a recent study, it was found that a Gγ protein regulated the phosphorylation of aquaporins. These aquaporins were channels that can transport hydrogen peroxide to help alleviate oxidative stress caused by salt stress [30]. Based on this finding, we investigated the expression patterns of genes related to aquaporins, kinases, and the scavenging of ROS during salt stress in both genotypes. A total of 21 aquaporin transcripts were detected in our dataset, including 11 PIP, 7 TIP, 2 NIP and 1 SIP family genes (Figure 4B). In both genotypes, 19 aquaporin genes showed low to high expression levels, especially showing an induced expression at the 72 h time point after salt stress. However, the expression levels of two TIP3-2 family genes were inhibited by salt stress in both genotypes (Figure 4B). These results indicated the diverging expression patterns of aquaporin genes in response to salt stress.

The process of protein phosphorylation was typically carried out by kinases [31]. We identified 24 kinase genes that responded to salt stress in our dataset, which included eight receptor-like protein kinase (RLK), four leucine-rich repeat receptor-like protein kinase (LRR-RLK), one proline-rich receptor-like protein kinase (PERK), two CBL (calcineurin B-like)-interacting serine/threonine-protein kinase 1 (CIPK1), five CIPK6, and four CIPK9 family genes (Figure 4C,D). When compared to the 0 h time point, the expression levels of eight RLK genes were induced by salt stress in both genotypes, with the highest levels observed at the 72 h time point (Figure 4C). Among the four LRR-RLK genes that positively responded to salt stress at different time points, the PXC2 gene was expressed much more highly in cv Jin-mian 25 than in Su-mian 3 and was induced at all four time points after salt stress in cv Jin-mian 25 (Figure 4C). Simultaneously, the expression level of a PERK9 gene decreased gradually with the duration of the salt stress in both genotypes, indicating that it negatively regulated salt stress (Figure 4C).

It has been reported in both Arabidopsis and cotton that CIPK6 kinase acts as a positive regulator of stress-responsive genes under salt stress [32,33,34]. The five CIPK6 genes identified here all exhibited the induced expression patterns at the 12, 24 or 72 h time points after salt stress treatment in both genotypes (Figure 4D). In contrast, the expression levels of the CIPK1 and CIPK9 genes were induced only at the 72 h time point of salt stress in both genotypes (Figure 4D).

ROS serve as signal molecules and participate in various plant physiological processes, but the ROS content must be kept relatively stable. Salt stress, however, causes the accumulation of active oxygen in plants, leading to oxidative stress [35]. When the accumulation of ROS occurs in plants, they initiate the antioxidant system to eliminate the accumulated ROS, thus maintaining the steady state of ROS levels under salt stress [36,37]. In our data, we identified 11 ROS-scavenging enzyme genes whose expression levels changed after salt stress, including one catalase (CAT), one glutathione S-transferase (GST), two superoxide dismutase (SOD), and seven peroxidase (POD) genes (Figure 4E). In comparison to the 0 h time point, the expression levels of these 11 ROS-scavenging enzyme genes were induced by salt stress in both genotypes and increased gradually with the prolongation of salt stress, indicating that these genes were positively regulated by salt stress (Figure 4E).

### 2.5. Expression Patterns of Genes Related to Ethylene Biosynthesis and Signaling During Salt Stress in Both Genotypes

It has been shown that phytohormones not only regulate plant growth and development under normal conditions but also mediate various environmental stresses, including salt stress, to help plants adapt to challenging conditions [38]. To investigate the genes related to hormone biosynthesis and signaling that are regulated during salt stress, we compared the expression levels of hormone biosynthesis and signaling genes between cv Jin-mian 25 and Su-mian 3 plants.

**Figure 5:**
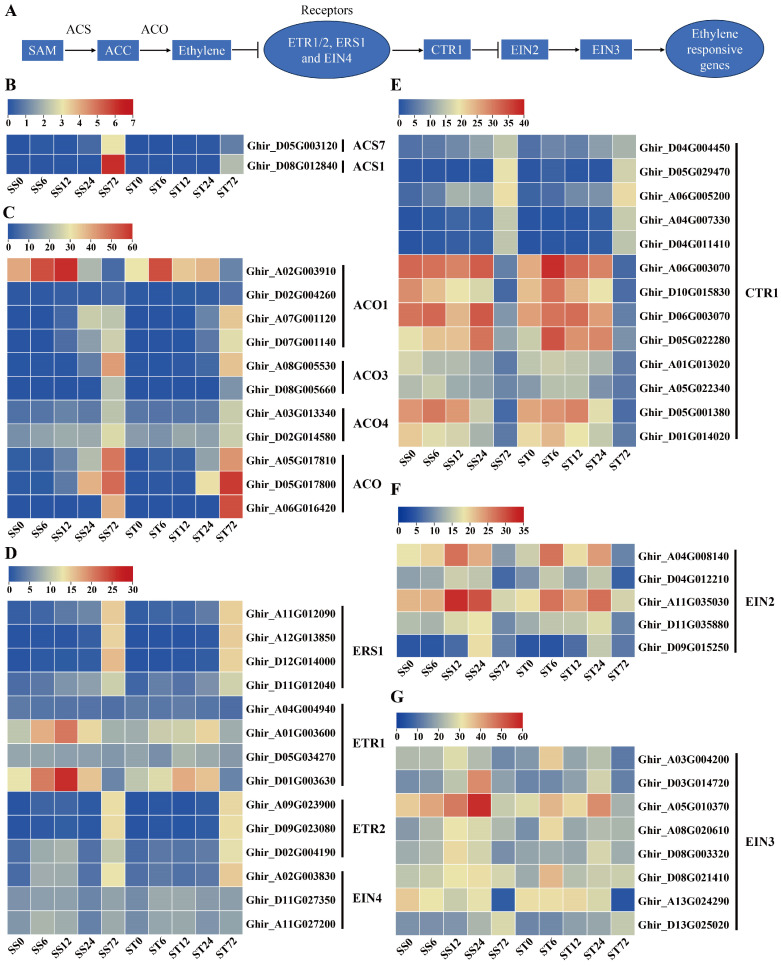
Expression of genes related to ethylene biosynthesis and signaling during salt stress. (**A**) Schematic overview of the ethylene biosynthesis and signaling pathway. Expression patterns of the ethylene biosynthesis genes in both genotypes during salt stress, including ACS genes (**B**) and ACO genes (**C**). Expression patterns of the ethylene signaling pathway genes in the two genotypes during salt stress, including receptors genes (**D**), inhibitor CTR1 genes (**E**), inhibitor EIN2 genes (**F**) and transcription factor EIN3 genes (**G**). Each row represents one gene, while the columns represent samples from different salt stress time points and the colors represent the gene expression levels as FPKM values. Lower levels of expression are represented in blue, higher expression is indicated in yellow, while the highest expression is indicated in red.

Previous studies proved that ethylene is biosynthesized from its precursor, S-adenosylMet (SAM), through two steps [39,40]. The first step involves the conversion of SAM to 1-aminocyclopropane-1-carboxylic acid (ACC) by aminocyclopropane-1-carboxylate synthase (ACS), while the second step is the conversion of ACC to ethylene by ACC oxidase (ACO) [41,42] (Figure 5A). Two ACS gene transcripts were detected in our dataset, showing an induced expression pattern at the 72 h time point after salt stress treatment in both genotypes, with higher transcripts in cv Su-mian 3 than in cv Jin-mian 25 (Figure 5B). In contrast, eleven ACO transcripts (four ACO1, two ACO3, two ACO4 and three ACO-like genes) were detected. In both genotypes, most ACO genes showed low to high expression levels, especially showing an induced expression at the 72 h time point after salt stress treatment, while one ACO1 was firstly high expressed at 6 and 12 h but showed very low levels at the 72 h time point (Figure 5C). The above data showed that the important enzymes ACS and ACO, which are involved in ethylene synthesis, might be crucial to the cotton plant’s response to salt stress.

The ethylene signal transduction process in the model plant Arabidopsis has been studied quite clearly [40]. The components of the ethylene signaling pathway include ethylene receptors (ETR1, ETR2, ERS1, ERS2 and EIN4) located on the endoplasmic reticulum, signal pathway inhibitors CTR1, EIN2, transcription factor EIN3/EIL1, downstream transcription factors and functional genes [43] (Figure 5A). A total of 14 ethylene receptors gene transcripts were detected. Among them, four ERS1, three ETR2 and one EIN4 membrane receptor genes only showed the induced expression pattern at the 72 h time point after salt stress treatment in both genotypes (Figure 5D). Two ETR1 genes presented high transcripts at 6, 12 and 24 h but low levels at the 72 h time point (Figure 5D). In addition, the ethylene signaling pathway inhibitor CTR1 genes showed divergent expression profiles after salt stress, among which five genes were induced, while the other eight genes were repressed only at the 72 h time point (Figure 5E). In contrast, most EIN2 and EIN3 gene transcripts was induced at the 6, 12 or 24 h time point but reduced after 72 h of salt stress treatment in both genotypes (Figure 5F,G). Generally, the expression patterns of the genes involved in ethylene biosynthesis and signaling were mostly similar in both the cv Su-mian 3 and Jin-mian 25 plants (Figure 5), which indicated that the ethylene signaling pathway was involved in the salt stress response in both genotypes.

### 2.6. Expression Patterns of Genes Related to Gibberellin (GA) Biosynthesis and Signaling During Salt Stress in Both Genotypes

**Figure 6:**
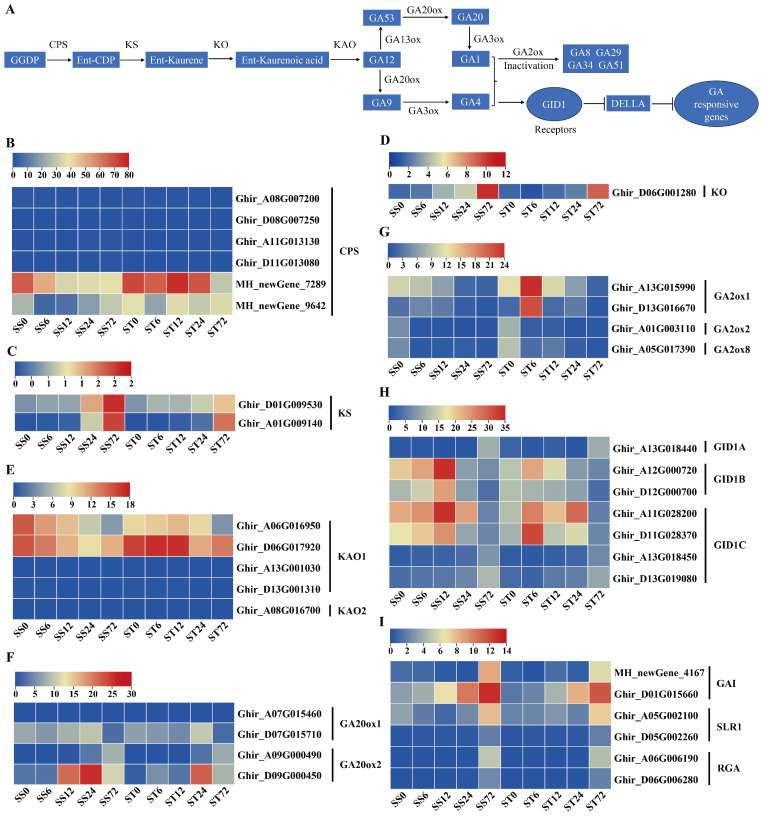
Expression of genes related to GA biosynthesis and signaling during salt stress. (**A**) Schematic overview of the GA biosynthesis and signaling pathway. Expression patterns of the GA biosynthesis genes in both genotypes during salt stress, including CPS genes (**B**), KS genes (**C**), KO genes (**D**), KAO genes (**E**), GA20ox genes (**F**). Expression patterns of the GA signaling pathway genes in two genotypes during salt stress, including GA2ox genes (**G**), receptor GID genes (**H**), and inhibitor DELLA genes (**I**). Each row represents one gene, the columns represent samples from different salt stress time points and the colors represent the gene expression levels as FPKM values. Lower levels of expression are represented in blue, higher expression is indicated in yellow, while the highest expression is indicated in red.

GA was also involved in the salt stress response in plants [44]. The GA biosynthetic mutant *ga1*–*3* showed higher tolerance to high-salinity stress [45] and salinity stress would reduce the endogenous GA levels via upregulating the GA 2-oxidase gene [46]. The biosynthesis of gibberellins (GAs) begins with the transformation of trans-geranyl-geranyl diphosphate via four enzymatic steps to produce GA12 [47] (Figure 6A). GA12 is then converted into GA53 by the enzyme GA13 oxidase (GA13ox), and GA12 and GA53 can be transformed into GA9 and GA20, respectively, by following two different pathways catalyzed by GA20ox [48]. Finally, GA9 and GA20 are converted into the bioactive GA forms GA4 and GA1 by the enzyme GA3ox [49] (Figure 6A). In our data, six GA biosynthetic metabolic enzymes, including *ent*-copalyl diphosphate synthase (CPS), *ent*-kaurene synthase (KS), *ent*-kaurene oxidase (KO), *ent*-kaurenoic acid oxidase (KAO), gibberellin 20 oxidase (GA20ox), and GA 2-oxidase (GA2ox), were detected (Figure 6B–G).

Six CPS and five KAO enzyme genes were identified, and both were repressed at 72 h after salt stress in both genotypes (Figure 6B,E). Among the KS and KO enzyme genes, their transcripts showed a slight increase at 72 h after salt stress in both genotypes, but both were expressed at low levels (less than 2 fpkm for KS or 12 fpkm for KO) during salt stress (Figure 6C,D). The transcripts of four GA20ox genes were detected in our dataset, and one GA20ox1 gene was repressed at 72 h after salt stress, while two GA20ox2 genes was induced markedly during salt stress in both genotypes (Figure 6F). The bioactive form of GA was inactivated by GA2ox enzymes [50]. The transcripts of four GA2ox genes were detected, and the expression patterns slightly diverged between the two genotypes. Among the four putative GA2ox genes detected, two genes (GA2ox2 and GA2ox8) were repressed at all time p while another two (GA2ox1) were induced at 6h but repressed at the other time points during salt stress in cv Jin-mian 25 (Figure 6G). In contrast, all four GA2ox gene transcripts were repressed during salt stress in cv Su-mian 3 (Figure 6G). The transcripts of the GA13ox and GA3ox enzymes genes were not detected in our dataset.

We further investigated the expression profiles of GA signaling-related genes during salt stress and found that the GA receptor GID1 and the GA signal pathway inhibitor DELLA genes was expressed after salt stress in both genotypes. Seven GA receptor gene transcripts were detected. Among them, one GID1A and two GID1C receptor genes showed the induced expression pattern at the 72 h time point after salt stress treatment in both genotypes (Figure 6H). Two GID1B and another two GID1C genes presented higher transcripts at 6, 12 or 24 h but lower levels at 72 h compared with 0 h (Figure 6H). In addition, six GA signaling pathway inhibitor DELLA genes, including two GAI, two SLR1 and two RGA, showed the induced expression patterns at the 24 or 72 h time point after salt stress in both genotypes (Figure 6I). Generally, the expression patterns of the genes involved in GA biosynthesis and signaling were mostly similar in both the cv Su-mian 3 and Jin-mian 25 plants (Figure 6), which indicated both genotypes required reduced bioactive GA levels or signaling for plant salt stress tolerance.

### 2.7. Expression Patterns of Genes Related to Abscisic Acid (ABA) Biosynthesis and Signaling During Salt Stress in Both Genotypes

**Figure 7:**
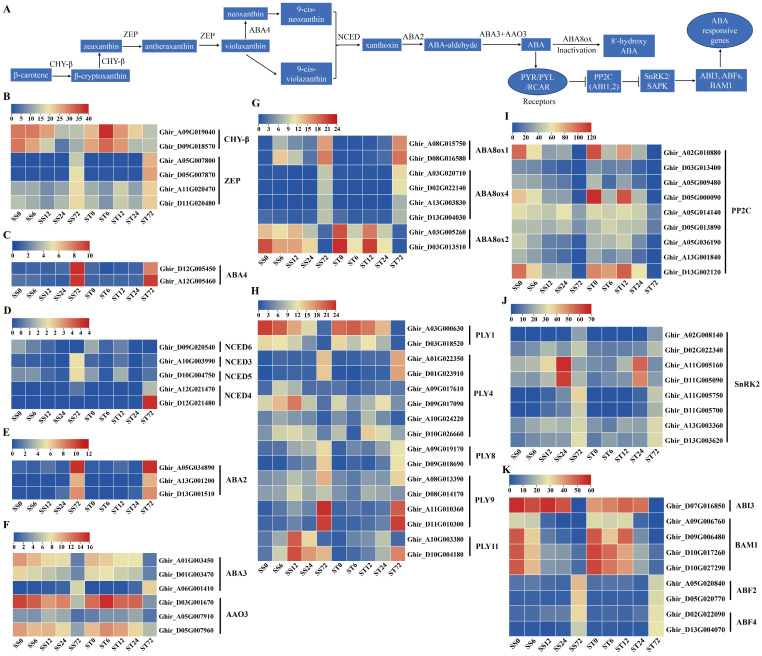
Expression of genes related to ABA biosynthesis and signaling during salt stress. (**A**) Schematic overview of the ABA biosynthesis and signaling pathway. Expression patterns of the ABA biosynthesis genes in both genotypes during salt stress, including CHY-β and ZEP genes (**B**), ABA4 genes (**C**), NCED genes (**D**), ABA2 genes (**E**), ABA3 and AAO3 genes (**F**). Expression patterns of the ABA signaling pathway genes in two genotypes during salt stress, including ABA8ox genes (**G**), PLY receptor genes (**H**), PP2C genes (**I**), SnRK2 genes (**J**), and ABI3, ABFs or BAM1 genes (**K**). Each row represents one gene, the columns represent samples from different salt stress time points and the colors represent the gene expression levels as FPKM values. Lower levels of expression are represented in blue, higher expression is indicated in yellow, while the highest expression is indicated in red.

ABA is a growth inhibitor with an imposition by salt stress [51]. In higher plants, β-carotene, the precursor of ABA, is synthesized via the plastidial methylerythritol phosphate (MEP) pathway from isopentenyl pyrophosphate [52,53]. Then, β-carotene is catalyzed by six enzymes and eventually converted into ABA through several steps (Figure 7A). In our data, seven ABA biosynthetic metabolic enzymes, including β-carotene hydroxylase (CHY-β), zeaxanthin epoxidase (ZEP), neoxanthin synthase (NSY/ABA4), 9-*cis*-epoxycarotenoid dioxygenase (NCED), xanthoxin dehydrogenase (ABA2), molybdenum cofactor sulfurase (ABA3) and abscisic-aldehyde oxidase (AAO3), were detected (Figure 7A).

Two CHY-β enzyme genes were identified and both were repressed at 24 or 72 h after salt stress in both genotypes (Figure 7B). Among the four ZEP and two ABA4 genes, their transcripts were induced markedly at 72 h after salt stress in both genotypes (Figure 7B,C). NCED is a rate-limiting enzyme in ABA biosynthesis [54], and five NCED genes were detected in our dataset, but all were expressed at low levels (less than 4 fpkm) during salt stress (Figure 7D). The transcripts of NCED3 and NCED5 showed a slight increase at 72 h after salt stress in cv Su-mian 3, while two NCED4 genes showed the upregulated transcripts at 72 h after salt stress in cv Jin-mian 25 (Figure 7D). The transcripts of three ABA2 genes were induced markedly during salt stress in both genotypes (Figure 7E). Among the three ABA3 and three AAO3 genes, only the expression of one ABA3 was induced at 72 h, but the other five gene transcripts were repressed at 72 h after salt stress in both genotypes (Figure 7F). ABA is inactivated by the action of ABA 8′-hydroxylase (ABA8Ox), which is one of the key steps involved in modulating the ABA level [55]. The transcripts of the ABA8ox1 and ABA8ox4 genes were substantially induced at 72 h of salt stress treatment (Figure 7G). Additionally, the expression of two ABA8ox2 genes was reduced at 72 h of salt stress treatment in both genotypes (Figure 7G).

The expression profiles of the ABA signaling pathway genes during salt stress were also investigated in both genotypes. A total of 16 ABA receptor gene transcripts were detected. Among them, two PLY1 receptor genes showed a reduced expression pattern at 72 h of salt stress treatment in both genotypes (Figure 7H). The PLY4, PLY8, PLY9 and PLY11 receptor genes presented induced transcripts at 6, 12, 24 or 72 h compared with 0 h (Figure 7H). Overall, the transcripts of the ABA receptor genes (PLY family) were induced by salt stress treatment. 

In addition, key players in ABA signaling transduction, including the PP2C, SnRK2, ABI3, BAM1, and ABFs genes, were also detected in our data. The transcripts of nine PP2C genes were suppressed at 12, 24 or 72 h after salt stress in both genotypes (Figure 7I). However, eight SnRK2 genes showed upregulated transcripts at 12, 24 or 72 h after salt stress in both genotypes (Figure 7J). We also found that ABI3 gene expression was repressed at 72 h of salt stress treatment in both genotypes (Figure 7K). In addition, four BAM1 genes showed reduced expression patterns after salt stress; on the contrary, two ABF2 and two ABF4 showed induced expression patterns at the 72 h time point after salt stress in both genotypes (Figure 7K). Generally, the genes involved in ABA biosynthesis and signaling were expressed similarly in both the cv Su-mian 3 and Jin-mian 25 plants (Figure 7). Our data indicated that the enhanced ABA-activated SnRKs signaling module might be involved in salt stress regulation in cotton plants. Meanwhile, ABA exhibited complicated signaling crosstalk to control cotton plant resistance against salt stress.

### 2.8. Expression Patterns of Genes Related to Jasmonic Acid (JA) Biosynthesis and Signaling During Salt Stress in Both Genotypes

**Figure 8:**
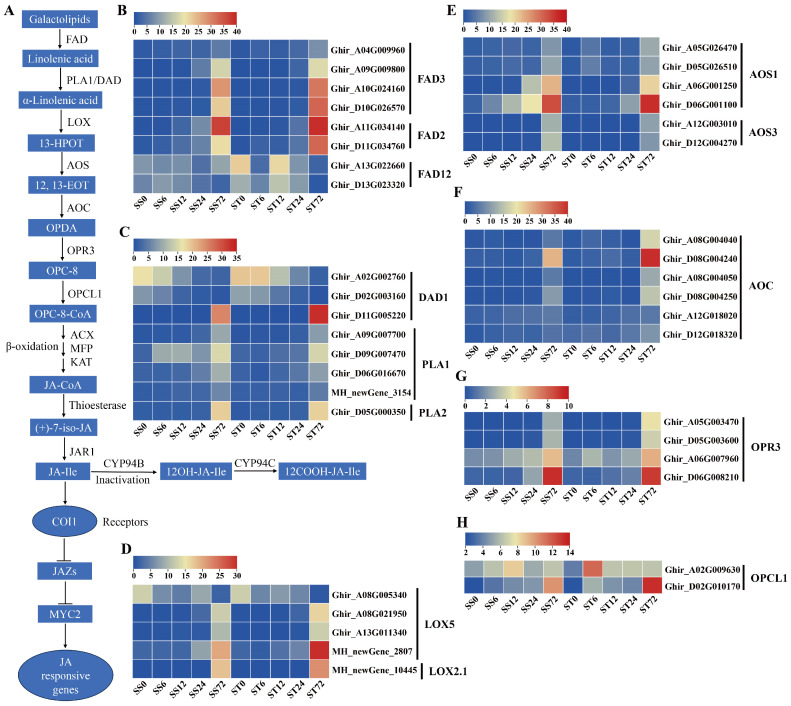
Expression of genes related to JA biosynthesis and signaling during salt stress. (**A**) Schematic overview of the JA biosynthesis and signaling pathway. Expression patterns of the JA biosynthesis genes in both genotypes during salt stress, including FAD genes (**B**), PLA1 and DAD1 genes (**C**), LOX genes (**D**), AOS genes (**E**), AOC genes (**F**), OPR3 genes (**G**), and OPCL1 genes (**H**). Each row represents one gene, the columns represent samples from different salt stress time points and the colors represent the gene expression levels as FPKM values. Lower levels of expression are represented in blue, higher expression is indicated in yellow and the highest expression is indicated in red.

JA controls the responses to salt stress tolerance in plants [56,57]. Jasmonates (JAs) originate from α-linolenic acid (Figure 8A), which is produced from chloroplast membrane galactolipids catalyzed by fatty acid desaturase (FAD) and phospholipase A1 (PLA1) or defective anther dehiscence 1 (DAD1) enzymes [58]. Then, several enzymes, including lipoxygenase (LOX), allene oxide synthase (AOS), allene oxide cyclase (AOC), 12-oxophytodienoic acid reductase 3 (OPR3), and OPC-8:0 CoA ligase 1 (OPCL1), convert α-linolenic acid to OPC-8-CoA [59,60], which subsequently undergoes three rounds of β-oxidation to yield jasmonoyl-CoA, which is then cleaved to (+)-7-iso-jasmonoyl by thioesterase (Figure 8A). The three key enzymes involved in β-oxidation are acyl-CoA oxidase (ACX), a multifunctional protein (MFP), and 3-keto-acyl-CoA thiolase (KAT). Finally, (+)-7-iso-jasmonoyl-Ile is produced by the catalyzation of the jasmonate-resistant 1 (JAR1) enzyme (Figure 8A). In our data, all 12 JA biosynthetic metabolic enzymes were detected (Figure 8A).

**Figure 9:**
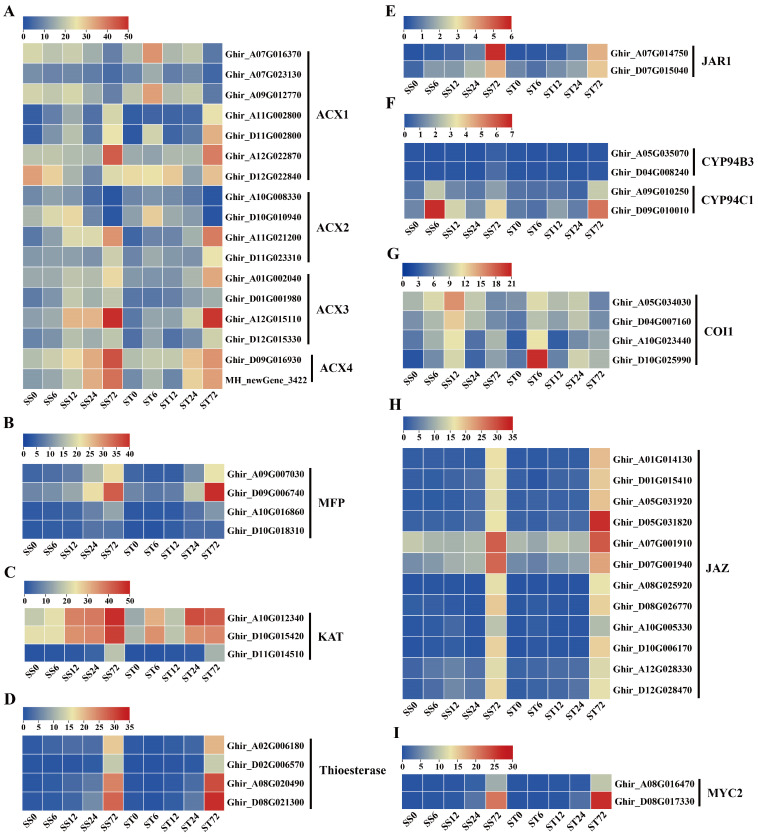
Expression of genes related to JA biosynthesis and signaling during salt stress. (**A**) Expression patterns of the JA biosynthesis genes in both genotypes during salt stress, including ACX genes (**A**), MFP genes (**B**), KAT genes (**C**), Thioesterase genes (**D**), and JAR1 genes (**E**). Expression patterns of the JA signaling pathway genes in two genotypes during salt stress, including CYP94 genes (**F**), COI1 genes (**G**), JAZ genes (**H**), and MYC2 genes (**I**). Each row represents one gene, the columns represent samples from different salt stress time points and the colors represent the gene expression levels as FPKM values. Lower expression levels are represented in blue, higher expression is indicated in yellow and the highest expression is indicated in red.

Eight FAD enzyme genes were identified. Among them, the expression levels of four FAD3 and two FAD2 were markedly induced at 72 h, while two FAD12 were repressed at 72 h after salt stress in both genotypes (Figure 8B). Among the four PLA1 and three DAD1 genes, most of their expression levels were induced markedly at 72 h after salt stress in both genotypes (Figure 8C). Five LOX genes were detected in our dataset, and the expression levels of four LOX genes showed a significant increase at 72 h after salt stress in both genotypes, while one LOX5 gene showed downregulated expression at 72 h after salt stress (Figure 8D). The transcripts of the AOS (Figure 8E), AOC (Figure 8F), OPR3 (Figure 8G) and OPCL1 (Figure 8H) genes were all induced markedly during salt stress in both genotypes. Among the 17 ACX genes, 12 of their expression levels (four ACX1, two ACX2, four ACX3, and two ACX4) were induced during salt stress, but the other five ACX1 and ACX2 gene expressions were repressed at 72 h after salt stress in both genotypes (Figure 9A). The transcripts of the MFP (Figure 9B), KAT (Figure 9C), thioesterase (Figure 9D) and JAR1 (Figure 9E) genes were all induced markedly during salt stress in both genotypes. In summary, the expression levels of the JA biosynthetic metabolic enzyme genes were induced by salt stress treatment in both genotypes. JA-Ile is inactivated by the activity of members of the CYP94 family through hydroxylation (Figure 8A). Two CYP94B3 genes detected were not expressed (less than 1 fpkm) during salt stress (Figure 9F). The expression levels of two CYP94C1 showed a slight increase at 72 h after salt stress in both genotypes, but both were expressed at low levels (less than 7 fpkm) (Figure 9F).

The expression profiles of the JA signaling pathway genes were also investigated during salt stress in both genotypes. COI1 and JAZ co-receptor gene transcripts were detected. Four COI1 receptor genes showed an induced expression pattern during salt stress treatment in both genotypes (Figure 9G), and 12 JAZ protein genes presented significantly induced expression profiles at 72 h compared with 0 h (Figure 9H). Overall, the expression levels of the JA co-receptor genes were induced by salt stress treatment in both genotypes. In addition, MYC2 genes, as the key transcription factor involved in JA signaling transduction, were also detected in our data. The expression levels of two MYC2 genes were higher at 72 h after salt stress in both genotypes (Figure 9I). Generally, the genes involved in JA biosynthesis and signaling were expressed similarly in both the cv Su-mian 3 and Jin-mian 25 plants (Figure 8 and Figure 9), which indicated that the JA biosynthesis and signaling pathways were positively involved in the salt stress response in both genotypes.

### 2.9. Expression Patterns of Genes Related to Brassinosteroids (BRs) Biosynthesis and Signaling During Salt Stress in Both Genotypes

**Figure 10:**
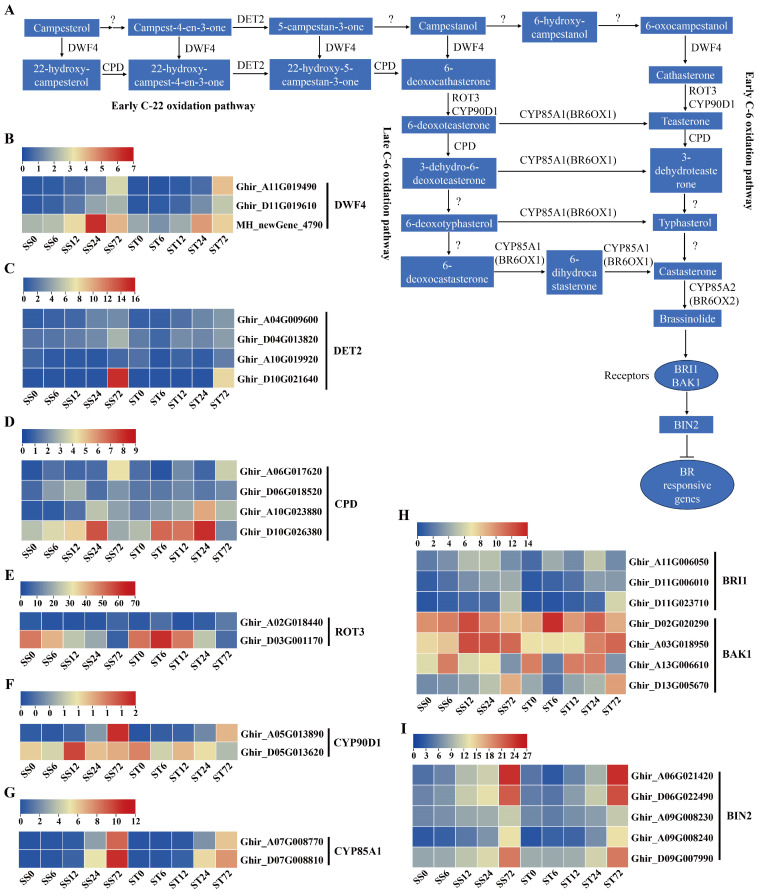
Expression of genes related to BR biosynthesis and signaling during salt stress. (**A**) Schematic overview of the BR biosynthesis and signaling pathway. Expression patterns of the BR biosynthesis genes in both genotypes during salt stress, including DWF4 genes (**B**), DET2 genes (**C**), CPD genes (**D**), ROT3 genes (**E**), CYP90D1 genes (**F**), and CYP85A1 genes (**G**). Expression patterns of the BR signaling pathway genes in two genotypes during salt stress, including BRI and BAK1 receptor genes (**H**), and BIN2 genes (**I**). Each row represents one gene, the columns represent samples from different salt stress time points and the colors represent the gene expression levels as FPKM values. Lower levels of expression are represented in blue, higher expression is indicated in yellow and the highest expression is indicated in red.

BRs are a type of steroid phytohormones found in plants, and it has been widely reported that BRs can enhance salt stress tolerance in various plants [61,62]. The detailed study of the biosynthesis of brassinolide, which is a C28 BR, showed that there are two parallel pathways, the early and late C-6 oxidation pathways [63,64]. These pathways are interconnected at multiple steps and are also linked to the early C-22 oxidation pathway [65,66,67]. Therefore, the biosynthetic pathways of BR are highly connected. Additionally, information about the enzymes and genes involved in BR biosynthesis, as well as their regulation, has been identified, including CYP90A1 (CPD), CYP90B1 (DWF4), CYP90C1 (ROT3), CYP90D1, DET2, brassinosteroid-6-oxidase 1/2 (BR6OX1/2, also known as CYP85A1/2), membrane-localized receptors BRASSINOSTEROID-INSENSITIVE 1 (BRI1), and the coreceptor BAK1 (Figure 10A).

Three DWF4 enzyme genes were identified and their expression levels were markedly induced at 24 or 72 h after salt stress in both genotypes (Figure 10B). Among the four DET2 genes, most of their expression levels were induced markedly at 72 h after salt stress in both genotypes (Figure 10C). Four CPD genes were detected in our dataset, and their expression levels showed a significantly increase at 24 or 72 h after salt stress in both genotypes (Figure 10D). Among the two ROT3 genes, one of the expression levels was induced, but the other one was repressed during salt stress in both genotypes (Figure 10E). The transcripts of the CYP90D1 (Figure 10F) and CYP85A1 (Figure 10G) genes were all induced slightly during salt stress in both genotypes and expressed at low levels (less than 12 fpkm). In summary, the expression levels of the BR biosynthetic metabolic enzyme genes were induced by salt stress treatment in both genotypes. 

The expression profiles of the BR signaling pathway genes were also investigated during salt stress in both genotypes. BRI1 and BAK1 co-receptor gene transcripts were detected. Three BRI1 receptor genes showed an induced expression pattern during salt stress treatment in both genotypes (Figure 10H), and only one BAK1 gene presented significantly induced expression profiles at 72 h compared with 0 h (Figure 10H). Overall, the expression levels of the BR receptor genes were induced by salt stress treatment in both genotypes. In addition, BIN2 genes, as the critical negative component of BR signaling, were also detected in our data. The expression levels of five BIN2 genes were higher at 12, 24 or 72 h after salt stress in both genotypes (Figure 10I). Generally, the genes involved in BR biosynthesis and signaling were expressed similarly in both the cv Su-mian 3 and Jin-mian 25 plants (Figure 10), which indicated that the BR biosynthesis and signaling pathways were positively involved in the salt stress response in both genotypes.

We conducted a systematic investigation into the expression patterns of genes related to five phytohormone biosynthesis and signaling pathways in both cv Su-mian 3 and Jin-mian 25 under salt stress. Despite the involvement of these hormones in cotton’s response to salt stress in both positive or negative ways, the expression levels of these genes in both genotypes are similar. Therefore, these genes are not the genetic basis of the divergent salt tolerance in both genotypes.

### 2.10. Expression Patterns of Genes Related to Trehalose Metabolism Pathway During Salt Stress in Both Genotypes

**Figure 11:**
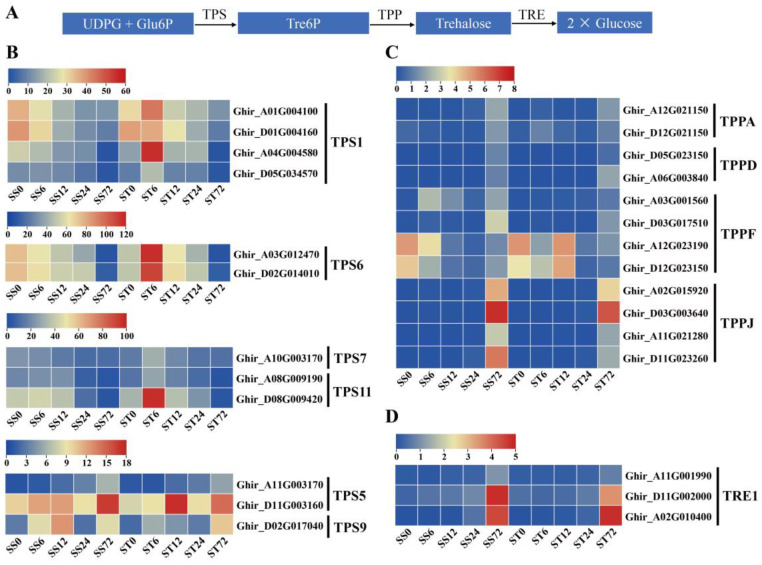
Expression of genes related to trehalose metabolism during salt stress. (**A**) Schematic overview of the trehalose biosynthesis and degradation pathway. Expression patterns of the trehalose biosynthesis and degradation genes during salt stress in both genotypes, including TPS genes (**B**), TPP genes (**C**), and TRE1 genes (**D**). Each row represents one gene, the columns represent samples from different salt stress time points and the colors represent the gene expression levels as FPKM values. Lower levels of expression are represented in blue, higher expression is indicated in yellow and the highest expression is indicated in red.

We delved further into the expression of genes in other relevant pathways to comprehend the metabolic adaptation of salt-tolerant cotton under salt stress [68,69]. As a disaccharide that functions in carbohydrate transport and metabolism, trehalose alleviates salt stress in plants by regulating the trehalose metabolic pathway [70,71]. In plants, trehalose is produced from UDP-Glc and Glc-6-P by trehalose-6-phosphate synthases (TPS) and trehalose-6-phosphate phosphatases (TPP), and it is degraded by trehalase (TRE) (Figure 11A).

In our dataset, the expression levels of 12 TPS, 12 TPP, and three TRE genes were detected. Notably, nine TPS genes (four TPS1, two TPS6, one TPS7, and two TPS11) were strongly induced at the 6 h time point after salt stress in the cv Jin-mian 25 plants but not in the cv Su-mian 3 plants (Figure 11B). Three TPS genes (two TPS5, and one TPS9) were markedly induced in response to salt stress in both genotypes (Figure 11B). Moreover, 10 of the 12 TPP genes showed elevated expression levels at the 72 h time point in response to salt stress, with the expression at relatively low levels in both genotypes (Figure 11C). The expressions of three TRE1 genes were also upregulated after salt stress, and the expression patterns were similar in both genotypes (Figure 11D).

## 3. Discussion

Salt stress is a significant environmental factor that impacts the growth and development of plants [72]. As plants are immobile, they must develop appropriate mechanisms to adapt to high-salt environments [73]. Salt stress significantly affects plant growth and development, with significant inhibition of seed germination. In this study, cotton seeds were exposed to an increasing duration of salt stress and the growth of Su-mian 3 (salt-sensitive) was more severely inhibited than that of Jin-mian 25 (salt-tolerant). This study aimed to understand the transcriptional responses associated with salt stress in the salt-tolerant cotton cv Jin-mian 25 and the salt-sensitive cotton cv Su-mian 3. Cataloging the entire transcriptome using an RNA-Seq approach revealed differences in gene expression between salt-tolerant cotton (cv Jin-mian 25) and salt-sensitive cotton (cv Su-mian 3) plants, as well as unique and common responses to salt stress in both genotypes. The results of our thorough analysis of the transcriptome contribute to the discovery of key genes that regulate metabolic pathways during salt stress in cotton.

Previous studies have demonstrated that the phytohormones, including ethylene (ET), gibberellin (GA), abscisic acid (ABA), jasmonic acid (JA), and brassinosteroid (BR), modulate salt stress resistance in plants [38,44]. Ethylene signaling can influence the salt response at various levels, including membrane receptors, cytoplasmic components, and nuclear transcription factors within the pathway [74,75]. Three gain-of-function mutations of the ethylene receptors *CpETR1B*, *CpETR1A*, and *CpETR2B* in *Cucurbita pepo* all reduced ethylene sensitivity but enhanced salt tolerance during seed germination and plant growth [76]. The enhanced salt tolerance response of the mutants was associated with the change of cytoplasmic components, presenting a reduced accumulation of Na^+^ but a higher accumulation of proline, total carbohydrates, and anthocyanin. In addition, the expression levels of many membrane transporter genes, including Na^+^/H^+^ and K^+^/H^+^ exchangers, K^+^ efflux antiporters, high-affinity K^+^ transporters, and K^+^ uptake transporters, were significantly upregulated under salt stress in the mutants in comparison with the WT [76]. Expression data indicated that two tomato *ERFs* were more strongly induced in salt-tolerant genotypes than in salt-sensitive genotypes, and over-expressing *SpERF.B7* in Arabidopsis exhibited enhanced salt tolerance, proving that ERF transcription factors were involved in the salt response in tomato [77].

Many reports indicated that ethylene and related components were involved in the plant salt stress response via the clearance of ROS [78]. For example, ethylene can improve *Arabidopsis thaliana*’s tolerance to salinity by regulating RBOHF-mediated ROS and Na/K balance [79]. Ethylene can also stabilize EIN3/EIL1 to enhance the expression of SIEDs and PODs, thereby clearing the accumulation of ROS in plant cells [80]. Meanwhile, ethylene treatment can recover the germination rate of alfalfa seeds under salt stress by reducing the accumulation of MDA and H_2_O_2_ and increasing the POD activity [81]. A recent study also showed that at 150 mM NaCl stress, 2 mM ethephon treatment significantly increased the germination rate and potential of cotton seeds by more than 70%. Subsequent evidence proved that exogenous ethylene improved the salt tolerance and germination rate of cotton seeds by boosting the antioxidant enzyme activity, increasing the proline content, and reducing membrane lipid peroxidation [82]. These findings suggest that ethylene can mediate the balance of ROS in plant cells to affect plant salt tolerance.

By contrast, the GA-deficient mutant exhibited remarkable tolerance to salt stress [45]. Consistently, salt-stress-induced plant DELLA accumulation elevated the expression levels of genes encoding ROS-detoxification enzymes to reduce the ROS levels, thus delaying cell death and promoting tolerance [83]. In essence, these results indicated that reduced bioactive GA levels are required for plant tolerance to salt stress. The ABA and GA signaling pathways appear to be interacting in the regulation of seed germination and seedling development under salt stress [84]. *OsNCED5*, a rice NCED gene, was induced by exposure to salt stress. Overexpression of *OsNCED5* increased the ABA levels and enhanced salt stress tolerance, while *nced5* mutants reduced the ABA levels and decreased tolerance to salt stress. Therefore, *OsNCED5* might regulate plant development and salt stress resistance by controlling ABA biosynthesis [54]. Thus, the endogenous ABA levels increased under salinity stress in plants and enhanced ABA signaling activated SnRK2s [85]. SnRK2s played critical roles in ROS production in ABA signaling networks [86]. Indeed, two SnRK2 kinases, SnRK2.4 and SnRK2.10, were involved in the regulation of ROS homeostasis in response to salinity in *A. thaliana* [87]. Together, ABA and ROS exhibit close signaling crosstalk to regulate plant resistance to salt stress.

In Arabidopsis, JA impaired plant salt stress tolerance by repressing *Catalase2* expression, which led to lower CAT activity and higher ROS accumulation [57]. In wheat, exogenous JA was found to enhance the salt tolerance of wheat seedlings by reducing membrane lipid oxidation, upregulating the expression levels of antioxidative defense system genes, and increasing the activities of SOD, POD, CAT and APX [56]. Our analysis of the transcript indicates that salt stress triggers the expression of JA biosynthesis-related genes. Application of JA exogenously significantly alleviates salt-induced damage by increasing the antioxidative enzyme activities and maintaining the Na^+^/K^+^ balance [88,89]. As a stress-related hormone, JA signaling has been proven to play an essential role in plant salt tolerance.

BRs have also been widely reported to enhance salt stress tolerance in various plants [90], such as tomato [91], rice [92], and mustard [93]. Exogenous BR application decreased the Na^+^ accumulation and increased the K^+^ content to relieve salt toxicity by regulating the expression levels of Na^+^(K^+^)/H*^+^* antiporter genes [94]. Under saline conditions, BR can greatly reduce the generation of ROS by increasing antioxidant capacity [94,95]. These results highlight the potential roles of BR-mediated ROS homeostasis in plant salt resistance.

In cotton, significant progress has been made in understanding the genetic basis of salt tolerance in the last 10–15 years. Numerous salt-responsive genes have been identified by using genomic or transcriptomic methods or transgenic approaches for the enhancement of salt stress tolerance [96,97]. RNA-Seq experiments showed that cotton *GRF* genes exhibited decreased expression in leaves under NaCl treatment [97]. Aquaporin genes in response to salt stresses also were identified by transcriptome analysis. *GhPIP2;7*-silenced plants exhibited decreased SOD and POD activity under 400 mM NaCl treatment. *GhTIP2;1*-overexpressed plants showed reduced H_2_O_2_ and malondialdehyde accumulation but higher proline content under salt stress [98]. *GhSOS1*, a plasma membrane Na+/H+ antiporter gene from upland cotton, could enhance salt tolerance in transgenic *Arabidopsis thaliana* [99]. A cotton DRE-binding transcription factor gene, *GhDREB*, conferred enhanced tolerance to high salt stress in transgenic wheat [100]. A recent study used VIGS to silence the *GhSAMC* gene in cotton, which resulted in increased sensitivity to salt stress, with more ROS accumulation in leaves than in the control, suggesting that *GhSAMC* regulates ROS accumulation to enhance cotton’s salt tolerance [101]. For other plants, *GmGSTU23*, a tau-like glutathione transferase family gene, mediated the scavenging of ROS by enhancing the activity of glutathione transferase, thus conferring enhanced tolerance to salt stress in *Glycine max* [37]. Moreover, trehalose also enhanced the antioxidant activities and the expression of stress-responsive proteins and genes, providing salt tolerance in plants [102,103]. Furthermore, a G protein γ subunit involved in the salt response mainly affected the phosphorylation of aquaporins to modulate the distribution of H_2_O_2_ in sorghum, millet, rice, and maize [30]. It is evident that exposure to salt stress can cause plant cells to accumulate large amounts of ROS, necessitating an increase in the presence of reducing enzymes and substances to eliminate them.

## 4. Materials and Methods

### 4.1. Plant Materials and Experiment Design

The salt-tolerant cotton Jin-mian 25 and salt-sensitive cotton Su-mian 3 were used in this study. Plump cotton seeds that were evenly sized were selected and sterilized by soaking in 10% hydrogen peroxide for 30 min. Then, they were rinsed with sterile water 4–5 times and filter paper was used to absorb moisture from the seed surface. Then, the seeds from both genotypes were selected and treated for 0 h, 6 h, 12 h, 24 h, and 72 h with 150 mM NaCl solution. The seeds or the seedlings were collected for transcriptome analyses at the corresponding treatment time. All the samples (30 samples) were frozen in liquid nitrogen and stored at −80 °C. Three biological replicates were performed in this study. In addition, earlier time points (such as 6 h or 12 h) were selected to capture the initial rapid responses to salt stress, while later points (such as 24 h or 72 h) were selected to reveal more long-term adaptive mechanisms. 

### 4.2. Measurement of Phenotypic Parameters

The cotton seeds were placed parallelly on the rectangular filter papers soaked in distilled water and a 150 mM NaCl solution, respectively. Then, another soaked filter paper was placed on top of the seeds and folded in half to roll it into a cylinder, with this process repeated three times and the bottom tied with an elastic band. The cylinders were placed in a germination box filled with 3 cm of salt solution, with the control group being distilled water. The germination box was placed in the seed germination chamber, with a temperature of 30 ± 1 °C and relative humidity of 60%, under 12 h/12 h light/dark (light intensity ~16,000 µmol/m^2^/s) cycle. The germination potential and germination rate of the seeds were calculated on days 3 and 7. On the seventh day, five seedlings were randomly selected to calculate the fresh weight, seedling length, root length, and hypocotyl length, with each treatment repeated three times.

### 4.3. RNA Extraction and Sequencing

The total RNA from 30 samples was extracted using the RNAprep pure plant kit (TIANGEN, Beijing, China) according to the manufacturer’s instructions. The Su-mian 3 and Jin-mian 25 seeds exposed to salt stress for 0 h, 6 h, 12 h, 24 h and 72 h were named SS0/ST0, SS6/ST6, SS12/ST12, SS24/ST24, and SS72/ST72, respectively. A total of 1 μg of purified mRNA was utilized for cDNA library construction using the NEBNext Ultra™ RNA Library Prep Kit for Illumina (NEB, Ipswich, MA, USA), in accordance with the manufacturer’s instructions. To begin, mRNA was purified from the total RNA using magnetic beads with poly-T oligos attached. Fragmentation of the mRNA was carried out under elevated temperature conditions with divalent cations, using the NEBNext First Strand Synthesis Reaction Buffer (5X). The first strand of cDNA was synthesized using random hexamer primers and M-MuLV Reverse Transcriptase. Subsequently, second-strand cDNA synthesis was performed with DNA Polymerase I and RNase H. The remaining overhangs on the DNA fragments were converted into blunt ends through exonuclease and polymerase activities. Following this, the 3′ ends of the DNA fragments were adenylated, and NEBNext Adaptors containing hairpin loop structures were ligated for hybridization. The AMPure XP bead system (Beckman Coulter, Beverly, MA, USA) was then employed to select cDNA fragments of approximately 300 bp. PCR amplification of the library was conducted using Universal PCR primers alongside an Index (X) Primer and Phusion High-Fidelity DNA Polymerase. Finally, the PCR products were purified using the AMPure XP kit (Beckman Coulter, Beverly, MA, USA), and the quality of the library was assessed with the Agilent Bioanalyzer 2100 system. All the libraries were sequenced on an Illumina NovaSeq 6000 (Illumina, San Diego, CA, USA) platform using 150 bp paired-end reads. Three biological replicates were performed. 

### 4.4. Transcriptome Data Analysis

The raw data in fastq format were processed with the FASTX-Toolkit (https://evomics.org/resources/software/genome-analysis-tools/fastx-toolkit/, accessed on 19 September 2019). Clean data (clean reads) were obtained by removing low-quality reads, reads containing adapters, and poly-N from the raw data. Concurrently, the Q30 and GC content of the clean data were calculated. All the subsequent analyses were based on high-quality clean data. Clean reads from each sample were then mapped to the reference genome (Ghirsutumv1.1_HAU-AD1_genome_v1.0_v1.1) for the cotton cultivar TM-1 [12] (https://www.cottongen.org/species/Gossypium_hirsutum/HAU-AD1_genome_v1.0_v1.1, accessed on 29 July 2020) using HISAT2 software v2.0.5 (parameter: hisat2 -p --rna-strandness RF --known-splicesite-infile -x --dta -1 -2) [104,105]. StringTie software v2.2.0 [105] was used to assemble the transcripts, including novel splice variants, to identify new differentially expressed genes and transcripts. The MH_newGene were new transcripts or new genes based on this method of de novo assembly of transcripts, which involved processing a large set of raw sequencing reads and creating lists of new gene transcripts. Reads with, at most, one mismatch were utilized to calculate the gene expression levels. The gene expression values were calculated following the method of a previous study [106]. The DEGs between two samples were identified using DESeq2 and presented using the fragments per kilobase of transcript per million fragments mapped (FPKM) [107]. The resulting *p* values were adjusted using Benjamini and Hochberg’s approach for controlling the false discovery rate (FDR). Genes with an adjusted *p* value < 0.01 and |log_2_fold change| ≥ 1 were defined as differentially expressed. The RNA-Seq raw reads generated were available from the Genome Sequence Archive of the BIG Data Center of Sciences (https://bigd.big.ac.cn/) under accession number CRA017659.

### 4.5. Gene Functional Annotation Analyses

The functions of the DEGs were annotated using the following databases: Nr (NCBI nonredundant protein sequences, ftp://ftp.ncbi.nih.gov/blast/db/, accessed on 29 December 2024), Gene Ontology (GO) (Gene Ontology, http://www.geneontology.org/), and Kyoto Encyclopedia of Genes and Genomes (KEGG) (http://www.genome.jp/kegg/, accessed on 29 December 2024). The KEGG enrichment was analyzed using KOBAS 2.0 software [108]. GO analysis of the DEGs was carried out using the GOseq R package v4.4 [109].

### 4.6. Data Processing, Visualization, and Statistical Analysis

The bar diagrams were plotted using GraphPad Prism 6.0. The Venn diagram was drawn using an online tool (https://bioinformatics.psb.ugent.be/webtools/Venn/, accessed on 29 December 2024). Heat maps was drawn to display the gene expression patterns of the FPKM values by using the TBtools software v2.025 [110]. The R package available at https://www.r-project.org/ (accessed on 29 December 2024) was utilized for Student’s *t*-test. The Shapiro–Wilk test was used to assess the normality, confirming that the data followed a Gaussian distribution. The least significant difference (LSD) was employed to assess significance at either the 1% or 5% level. The analysis involved a minimum of three biological replicates for each sample.

## 5. Conclusions

We used comparative transcriptomics to analyze the DEGs in the two cultivars under salt stress to elucidate the molecular mechanisms of the differences in salt tolerance between Su-mian 3 and Jin-mian 25. Through assessment of the germination potential and germination rate of the seeds, as well as the fresh weight, seedling length, root length, and hypocotyl length under salt stress, Su-mian 3 was identified as a salt-sensitive cultivar, while Jin-mian 25 was a salt-tolerant cultivar. Then, transcriptome profiles of Su-mian 3 and Jin-mian 25 cotton seeds exposed to salt stress were determined. KEGG pathway enrichment analysis showed that the DEGs were mainly involved in plant hormone signal transduction metabolism. Jin-mian 25 had 10 unique DEGs induced by salt stress, which might confer the salt stress tolerance. Based on this finding, we investigated the expression patterns of genes related to aquaporins, kinases, and the scavenging of ROS during salt stress in both genotypes. Most aquaporins, kinases, and the scavenging of ROS genes showed an induced expression after salt stress. Moreover, the expression patterns of genes related to phytohormone ethylene biosynthesis and signaling during salt stress in both genotypes were also investigated. In this regard, ET, ABA, JA, and BR were positively involved in the salt stress response; however, reduced GA levels were required for salt stress tolerance in both genotypes. Our transcriptome analysis also showed that trehalose alleviated salt stress by regulating the trehalose metabolic pathway in both genotypes. These results indicated that complicated signaling crosstalk existed to control cotton’s resistance against salt stress. The candidate genes identified from the multiple metabolic pathways should be further examined to determine their roles in the responses of cotton plants to salt stress.

## Data Availability

The RNA-Seq raw reads generated are available from the Genome Sequence Archive of the BIG Data Center of Sciences (https://bigd.big.ac.cn/) under accession number CRA017659.

## Supplementary Materials

The following supporting information can be downloaded at https://www.mdpi.com/article/10.3390/ijms26010329/s1.

## Abbreviations

DEGs, differentially expressed genes; FPKM, fragments per kilobase of transcript per million mapped fragments; *G. hirsutum*, *Gossypium hirsutum*; *A. thaliana*, *Arabidopsis thaliana*; GO, Gene Ontology; KEGG, Kyoto Encyclopedia of Genes and Genomes; ROS, reactive oxygen species; ET, ethylene; GA, gibberellin; ABA, abscisic acid; JA, jasmonic acid; BR, brassinosteroid; LRR-RLK, leucine-rich repeat receptor-like protein kinase; cv, cultivar; POD, peroxidase; SOD, superoxide dismutase; CAT, catalase.
