# Supplementary material for: Time-Course Transcriptomics Analysis Reveals Molecular Mechanisms of Salt-Tolerant and Salt-Sensitive Cotton Cultivars in Response to Salt Stress
Source: Int J Mol Sci. 2025 Jan 2;26(1):329. doi: 10.3390/ijms26010329 (PMC11719879; doi:10.3390/ijms26010329)
Supplement: Supplementary file 1 [file ijms-26-00329-s001.zip › Table S1.pdf]

Table S1. Characteristics of the RNA-sequencing data from 30 samples of cotton.

Samples	Clean reads	Clean bases	GC Content (%)	%≥Q30
SS0-1	22,861,548	6,858,464,400	44.90	93.23
SS0-2	32,206,156	9,661,846,800	44.82	93.34
SS0-3	22,994,400	6,898,320,000	44.67	93.19
SS6-1	24,480,973	7,344,291,900	44.76	91.66
SS6-2	33,381,782	10,014,534,600	44.87	93.12
SS6-3	24,861,254	7,458,376,200	44.83	93.59
SS12-1	25,812,202	7,743,660,600	44.65	93.40
SS12-2	23,215,114	6,964,534,200	45.19	93.10
SS12-3	23,519,633	7,055,889,900	43.93	93.12
SS24-1	24,299,555	7,289,866,500	43.59	92.97
SS24-2	23,031,899	6,909,569,700	43.58	93.19
SS24-3	24,176,682	7,253,004,600	43.63	92.69
SS72-1	24,532,960	7,359,888,000	43.61	93.85
SS72-2	25,158,302	7,547,490,600	43.80	93.92
SS72-3	25,049,190	7,514,757,000	43.82	93.41
ST0-1	24,217,372	7,265,211,600	44.86	92.96
ST0-2	28,272,479	8,481,743,700	45.02	92.38
ST0-3	28,780,055	8,634,016,500	44.75	91.67
ST6-1	30,951,023	9,285,306,900	44.87	91.16
ST6-2	31,119,809	9,335,942,700	44.99	91.49
ST6-3	30,590,599	9,177,179,700	44.79	91.11
ST12-1	30,357,585	9,107,275,500	45.16	91.72
ST12-2	28,597,277	8,579,183,100	44.49	91.51
ST12-3	28,847,724	8,654,317,200	44.65	91.91
ST24-1	27,602,445	8,280,733,500	45.05	91.72
ST24-2	27,619,983	8,285,994,900	43.18	91.96
ST24-3	25,927,642	7,778,292,600	43.53	91.99
ST72-1	29,348,930	8,804,679,000	43.84	92.12
ST72-2	30,341,145	9,102,343,500	43.87	91.63
ST72-3	27,904,272	8,371,281,600	43.75	92.01

Note: Su-mian 3 is a salt-sensitive cultivar (SS-Salt Sensitive), and Jin-mian 25 is a salt-tolerant cultivar (ST-Salt Tolerant).
