# Supplementary material for: Time-Course Transcriptomics Analysis Reveals Molecular Mechanisms of Salt-Tolerant and Salt-Sensitive Cotton Cultivars in Response to Salt Stress
Source: Int J Mol Sci. 2025 Jan 2;26(1):329. doi: 10.3390/ijms26010329 (PMC11719879; doi:10.3390/ijms26010329)
Supplement: Supplementary file 1 [file ijms-26-00329-s001.zip › Table S2.pdf]

Table S2. The mapping results of RNA-seq clean reads from 30 cotton samples using the *G. hirsutum* genome of Ghirsutumv1.1_HAU-AD1_genome_v1.0_v1.1.

Sample	Reads aligned	Exonic	Intergenic	Intronic
SS0-1	41,427,547(90.61%)	32,574,120(82.76%)	6,377,127(10.95%)	2,476,300(6.29%)
SS0-2	58,964,314(91.54%)	46,281,433(82.59%)	9,216,678(11.22%)	3,466,203(6.19%)
SS0-3	42,106,345(91.56%)	33,126,259(82.69%)	6,393,464(10.85%)	2,586,622(6.46%)
SS6-1	44,095,402(90.06%)	34,582,242(82.49%)	6,643,537(10.67%)	2,869,623(6.84%)
SS6-2	60,329,366(90.36%)	47,812,463(83.32%)	8,983,876(10.52%)	3,533,027(6.16%)
SS6-3	44,989,060(90.48%)	35,455,220(82.86%)	6,778,009(10.7%)	2,755,831(6.44%)
SS12-1	46,030,088(89.16%)	35,940,129(82.26%)	7,320,187(11.4%)	2,769,772(6.34%)
SS12-2	40,727,213(87.72%)	31,920,704(82.6%)	6,297,376(10.9%)	2,509,133(6.49%)
SS12-3	44,347,288(94.28%)	34,987,118(82.79%)	6,803,897(11.16%)	2,556,273(6.05%)
SS24-1	46,310,494(95.29%)	35,377,137(80.33%)	7,955,918(12.91%)	2,977,439(6.76%)
SS24-2	43,777,462(95.04%)	34,084,239(81.87%)	7,034,684(11.74%)	2,658,539(6.39%)
SS24-3	45,829,890(94.78%)	35,317,600(81.1%)	7,683,298(12.4%)	2,828,992(6.5%)
SS72-1	47,519,018(96.85%)	37,897,029(83.43%)	7,097,148(11.01%)	2,524,841(5.56%)
SS72-2	48,696,325(96.78%)	39,405,920(84.83%)	6,820,743(9.86%)	2,469,662(5.32%)
SS72-3	48,101,593(96.01%)	38,848,132(84.72%)	6,782,793(9.89%)	2,470,668(5.39%)
ST0-1	44,350,353(91.57%)	34,986,181(82.83%)	6,702,238(10.86%)	2,661,934(6.3%)
ST0-2	51,606,448(91.27%)	40,717,001(83.09%)	7,952,642(10.92%)	2,936,805(5.99%)
ST0-3	52,575,893(91.34%)	41,385,709(82.65%)	7,966,032(10.91%)	3,224,152(6.44%)
ST6-1	55,240,582(89.24%)	43,276,732(82.79%)	8,688,373(10.94%)	3,275,477(6.27%)
ST6-2	54,210,107(87.10%)	42,264,239(82.64%)	8,680,995(10.97%)	3,264,873(6.38%)
ST6-3	54,886,857(89.71%)	43,232,905(83.07%)	8,355,585(10.6%)	3,298,367(6.34%)
ST12-1	54,165,126(89.21%)	42,862,986(83%)	8,216,860(11.02%)	3,085,280(5.97%)
ST12-2	53,132,109(92.90%)	42,287,888(83.54%)	7,732,703(10.31%)	3,111,518(6.15%)
ST12-3	52,493,354(90.98%)	41,621,540(83.25%)	7,675,588(10.36%)	3,196,226(6.39%)
ST24-1	49,111,181(88.96%)	38,931,406(83.2%)	7,164,033(10.35%)	3,015,742(6.45%)
ST24-2	53,056,741(96.05%)	40,637,838(80.27%)	8,863,427(12.71%)	3,555,476(7.02%)
ST24-3	49,341,646(95.15%)	37,773,462(80.37%)	8,454,765(13%)	3,113,419(6.62%)
ST72-1	56,005,463(95.41%)	44,990,548(84.37%)	8,142,491(10.25%)	2,872,424(5.39%)
ST72-2	58,027,280(95.62%)	46,666,690(84.43%)	8,306,029(10.04%)	3,054,561(5.53%)
ST72-3	53,990,655(96.74%)	43,890,186(85.21%)	7,369,378(9.49%)	2,731,091(5.3%)

Note: Su-mian 3 is a salt-sensitive cultivar (SS-Salt Sensitive), and Jin-mian 25 is a salt-tolerant cultivar (ST-Salt Tolerant).
