# Supplementary figures and images for: Time-Course Transcriptomics Analysis Reveals Molecular Mechanisms of Salt-Tolerant and Salt-Sensitive Cotton Cultivars in Response to Salt Stress
Source: Int J Mol Sci. 2025 Jan 2;26(1):329. doi: 10.3390/ijms26010329 (PMC11719879; doi:10.3390/ijms26010329)

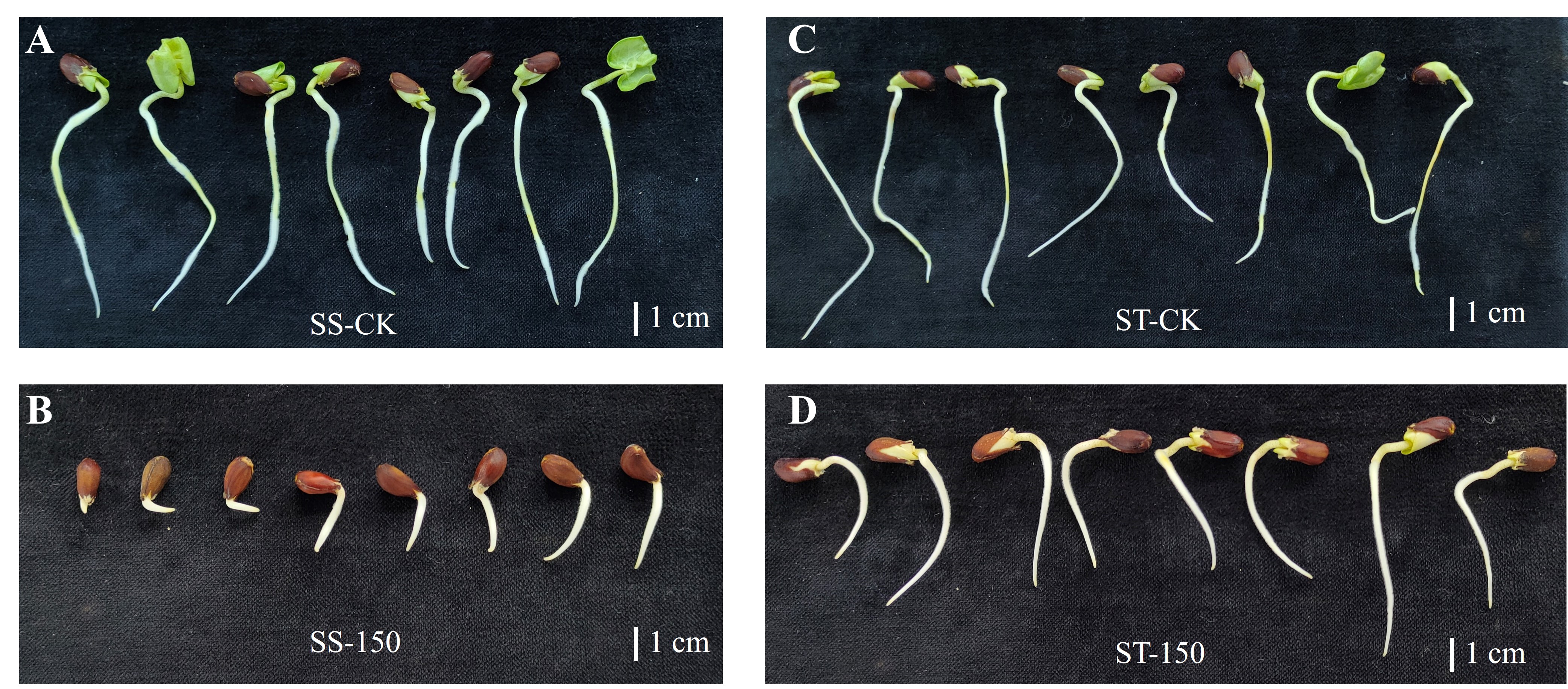

Supplement: Supplementary file 1 [file ijms-26-00329-s001.zip › Figure S1.jpg]
